# Identification of a *Shewanella halifaxensis* Strain with Algicidal Effects on Red Tide Dinoflagellate *Prorocentrum triestinum* in Culture

**DOI:** 10.3390/md21090501

**Published:** 2023-09-21

**Authors:** Victoria Cruz-Balladares, Vladimir Avalos, Hernán Vera-Villalobos, Henry Cameron, Leonel Gonzalez, Yanett Leyton, Carlos Riquelme

**Affiliations:** 1 Centro de Bioinnovación de Antofagasta, Universidad de Antofagasta, Antofagasta 1240000, Chilehernan.vera@uantof.cl (H.V.-V.); henry.cameron@uantof.cl (H.C.); carlos.riquelme@uantof.cl (C.R.); 2 Facultad de Ciencias del Mar y Recursos Biológicos, Universidad de Antofagasta, Antofagasta 1240000, Chile

**Keywords:** harmful algal blooms, *Prorocentrum triestinum*, bacterium supernatant, bioactive compounds, antagonist, algaecide

## Abstract

The dinoflagellate *Prorocentrum triestinum* forms high biomass blooms that discolor the water (red tides), which may pose a serious threat to marine fauna and aquaculture exploitations. In this study, the algicidal effect of a bacterial strain (0YLH) belonging to the genus *Shewanella* was identified and evaluated against *P. triestinum*. The algicidal effects on the dinoflagellate were observed when *P. triestinum* was exposed to cell-free supernatant (CFS) from stationary-phase cultures of the 0YLH strain. After 24 h exposure, a remarkable reduction in the photosynthetic efficiency of *P. triestinum* was achieved (55.9%), suggesting the presence of extracellular bioactive compounds produced by the bacteria with algicidal activity. Furthermore, the CFS exhibited stability and maintained its activity across a wide range of temperatures (20–120 °C) and pH values (3–11). These findings highlight the algicidal potential of the bacterium *Shewanella halifaxensis* 0YLH as a promising tool for the environmentally friendly biological control of *P. triestinum* blooms.

## 1. Introduction

Seawater discolorations, colloquially known as “red tides”, are caused by high concentrations of pigmented microalgae. Most of these high biomass algal blooms are harmless, but occasionally, if they are not consumed and decay, they become harmful. Negative impacts on the marine environment and aquaculture resources are caused by abrupt changes in physical-chemical conditions, leading to anoxia or hyperoxygenation, high concentrations of ammonium, the excretion of mucilaginous substances and even ecosystem disruption [1,2].

High Biomass Harmful Algal Blooms (HBHAB) are driven by a combination of abiotic and biotic factors, which creates optimal conditions for the rapid growth and/or accumulation of a particular species. Climate change may affect some of these factors, leading to increased seawater temperature and stratification and related changes in light intensity, turbulence and nutrients availability and composition [3,4,5].

Reports of water discolorations in northern Chile date back to the early XIX century, when Charles Darwin, in his travel diary aboard H.M.S. “Beagle”, described an intense coloration in the sea off Valparaiso and northern Concepcion [6]. Severe paralytic (PSP) and diarrhetic shellfish poisoning (DSP) outbreaks were first reported in the early 1970s, and were associated with high biomass blooms of toxic *Alexandrium catenella* and *Dinophysis acuta*, respectively [7,8]. These toxic HABs are mainly developed in the southern Patagonian provinces (reviewed by Avaria in [6]). In recent years, there has been an expansion of the HABs towards the north of Chile.

Recently, in Northern Chile, a high-density bloom persisted from November to February 2019, and occupied a huge marine area of 30 km across the shelf by 1500 km along the coast that stretched from the Atacama Region, Chile, to Arequipa, Perú. During this event, species of the genus *Prorocentrum* emerged as predominant, causing the massive death of squids and jellyfishes in Bahía Inglesa (27°7′ S; 70°52′ W, Atacama Region), probably associated with anoxia [9]. The genus *Prorocentrum* is a cosmopolitan dinoflagellate species with a complex life cycle, including benthic, planktonic and tychoplanktonic stages, and considerable morphological variability (size and shape) [10]. It is important to note that the genus *Prorocentrum* includes benthic species, and some of them are producers of diarrhetic shellfish toxins [11], e.g., *Prorocentrum lima* [12], and planktonic species. There are no toxic species within the planktonic *Prorocentrum*, but some of them (*P. micans, P. triestinum*) are frequent agents of HBHABs, particularly in upwelling systems [10,11].

Due to the negative impacts produced by HABs, various methods have been proposed to mitigate them [13]. Among these, there are physical methods which include the mechanical collection and removal of great amounts of microalgal biomass for its elimination; air pumping which produces microbubbles, generating flocs that move the microorganism to the surface for its removal [14]; magnetic separation, which uses mechanical energy together with iron oxide and powdered chloride to eliminate plankton [14,15]; separation by centrifugation; ultrasonic destruction, where sound waves are applied that affect the buoyancy of microalgae causing them to sink to the bottom of the water [14,15]; and ultraviolet radiation, which eliminates some species after a few seconds of exposure [16]. The disadvantage of physical methods is that they are slow and generate high operating costs. In addition, they are not very specific, thus affecting other aquatic organisms, so they are recommended only as a preventive strategy [14].

There are also chemical methods, such as clay flocculation, where flocs are formed by particle condensation, which promotes a rapid sinking of the aggregates. There are two types: natural clays, which have low efficiency and little flexibility, and more efficient modified clays, which are less expensive because lower quantities are required [14,17]. Another chemical method comprises surfactant compounds, which reduce the surface and interfacial tension between liquids, solids and gases. Surfactants, combined with clays, synergistically improve the removal of microalgae [14,15]. Lately, nanoparticles with algicides composed of titanium dioxide, zinc, cerium yttrium and aluminum oxides and structured barium titanate similar to coral have been designed, which act by trapping the cells and reducing the absorption of nutrients and photosynthesis [14].

Furthermore, there are widely distributed chemical products, such as ozone, chlorine, permanganate, copper sulfate, sodium hypochlorite and hydrogen peroxide, which can significantly inhibit microalgal growth, metabolic activity and photosynthesis. Their mechanism of action is based on the breakdown of cellular structures, which induces the production of free radicals, inhibits photosynthesis or hinders the synthesis of intracellular enzymes [15]. Although chemical methods are the most widely used, they are also the least safe for human health and aquatic environments. The toxicity they may have against other non-target organisms must be taken into consideration, and the releasing of residues into the environment should be reduced to the minimum [14].

The biological methods use either aquatic macroorganisms or microorganisms which produce bioactive compounds with algicidal activity. These methods are an effective alternative because they are less aggressive than the chemical and physical methods. Biological algicides have two ways of killing microalgae, either directly by contact between microorganisms with algicide activity and the algae or indirectly by exuding algicide compounds into the environment [13,14,15,18]. Among the algicidal producers, specific strains within the phylum *Actinomycetes* directly affect several bloom-forming species by inhibiting their physiological activity, inducing the production of reactive oxygen species (ROS) and causing oxidative damage [19]. Viruses also have potential as a biological control by causing lysis on cells, with the main advantage being the existence of a wide variety of species in the marine ecosystems, and also easy replication and a high specificity. However, there is a lack of information regarding virus–HABs interactions. In addition, parasitic pathogens are organisms that have been less studied, but are well known to recognize their prey, adhere to the surface, penetrate the cytoplasm, proliferate in the host and eventually cause cell death [14,15]. Finally, bacteria are the most studied microorganisms used as a biological control. Bacteria may act directly, a mode that requires bacteria-microalgae contact, inhibiting growth and causing cell lysis [14]. Conversely, indirect bacterial methods act by secreting bioactive compounds into the environment with negative effects on the microalgae, including changes in subcellular structure, the inhibition of photosynthesis and changes in enzymatic activity, functional gene expression and eventually cell lysis. The advantage of the compounds isolated from bacteria is that some of them are highly specific, a fact which turns them into a fairly safe and ecological control method against massive algae blooms [13,15,18,20,21]. Several bacteria belonging to the genera *Acinetobacter*, *Alcaligenes*, *Bacillus*, *Deinococcus*, *Hahella*, *Mangrovimonas*, *Pseudoalteromonas*, *Pseudomonas* and *Shewanella* have been extensively investigated for their ability to produce bioactive compounds that inhibit growth and lyse harmful algae species [19,21,22,23].

This work identified a new bacterial strain of the genus *Shewanella* with an apparent algicidal effect. The bioactive compounds were in the cell-free supernatant of the bacterial culture, affecting the cell morphology and photosynthetic activity of the dinoflagellate *Prorocentum triestinum*, leading to cell mortality in a short period of time.

## 2. Results

### 2.1. Characterization and Identification of the 0YLH Strain

**Figure 1:**
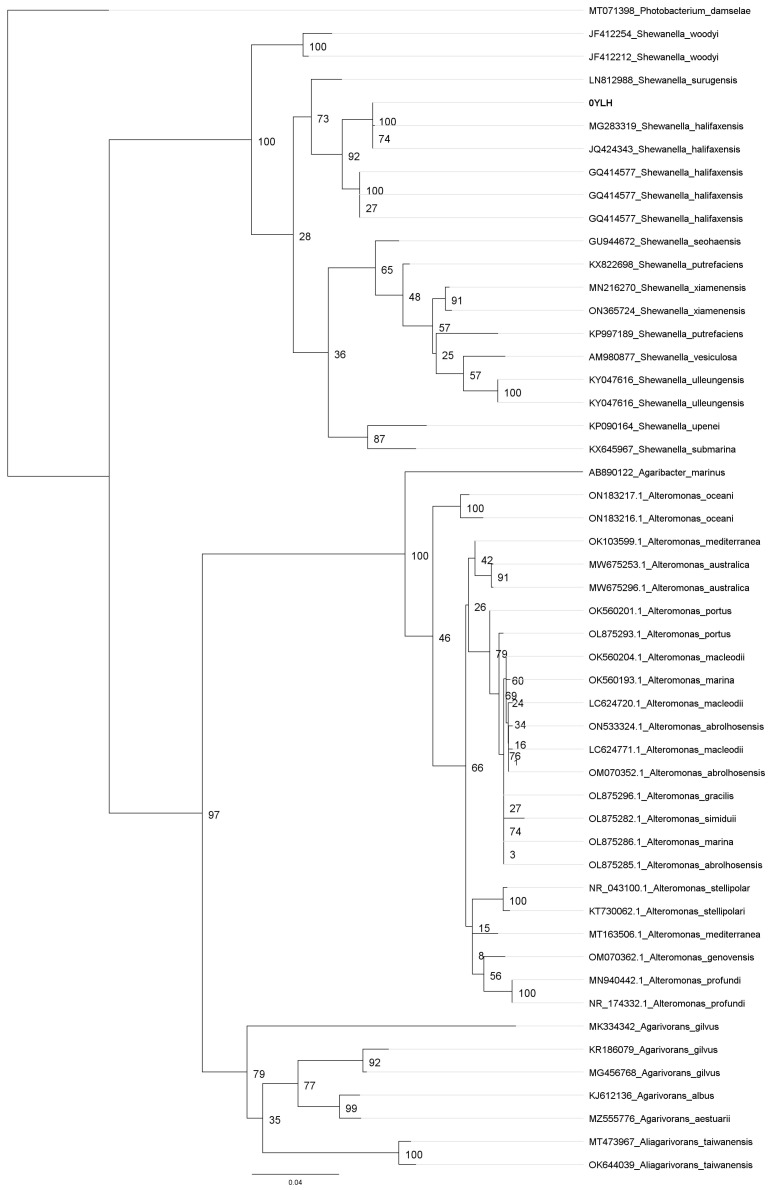
Phylogenetic tree of 16S rRNA gene of bacterial strain 0YLH. The phylogenetic tree was built through Tamura-Nei nucleotide substitution model with 100 bootstraps. Strain “0YLH” was aligned against 50 members of the Alteromonadales order. Outgroup used was *Photobacterium damselae* (accession number MT071398). PhyML algorithm was used on the MUSCLE alignment. Target bacterial sequence “0YLH” was highlighted with bold font.

The *Shewanella halifaxensis* strain 0YLH is a Gram-negative, rod-shaped and motile bacterium. The 16S rRNA phylogenetic tree analysis showed a visible separation between the Shewanellaceae and Alteromonadaceae families, both belonging to the order Alteromonadales. In this context, our bacterial isolate denoted as 0YLH was grouped inside the *S. halifaxensis* clade and was closely related with two other *S. halifaxensis* strains (accession number MG283319 and JQ424343) (Figure 1). In addition, the *S. halifaxensis* clade had a high degree of conservation since its bootstrap score was over 75.

### 2.2. Algicidal Activity

**Figure 2:**
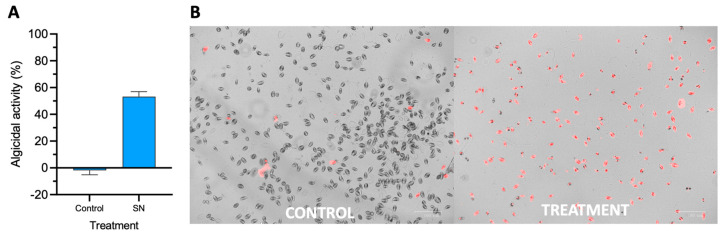
Antagonistic effect of strain 0YLH against the dinoflagellate *P. triestinum*. (**A**) Antagonist assay with 0YLH supernatant after 24 h, (**B**) cells stained with propidium iodide (PI) to determine cell killing activity (red coloration). Images were taken using the ZOE Fluorescent Cell Imager (BIO-RAD, Hercules, CA, USA).

The CFS secreted from the 0YLH strain exhibited significant antagonistic activity against *P. triestinum* when the dinoflagellate was treated at a 20% (*v*/*v*) ratio. This activity was reflected in the Fv/Fv parameter related to photosynthetic efficiency, which was drastically diminished reaching values near 0. Thus, this data allowed us to estimate the algicidal activity of the CFS. Notably, a reduction in photosynthetic efficiency was observed after 24 h of exposure, with 55.9% algicidal activity of the CFS, in contrast to the control, which exhibited negative values (Figure 2A). Our results showed a high predominance of PI fluorescence when *P. triestinum* was exposed to CFS as compared to the control (Figure 2B).

**Figure 3:**
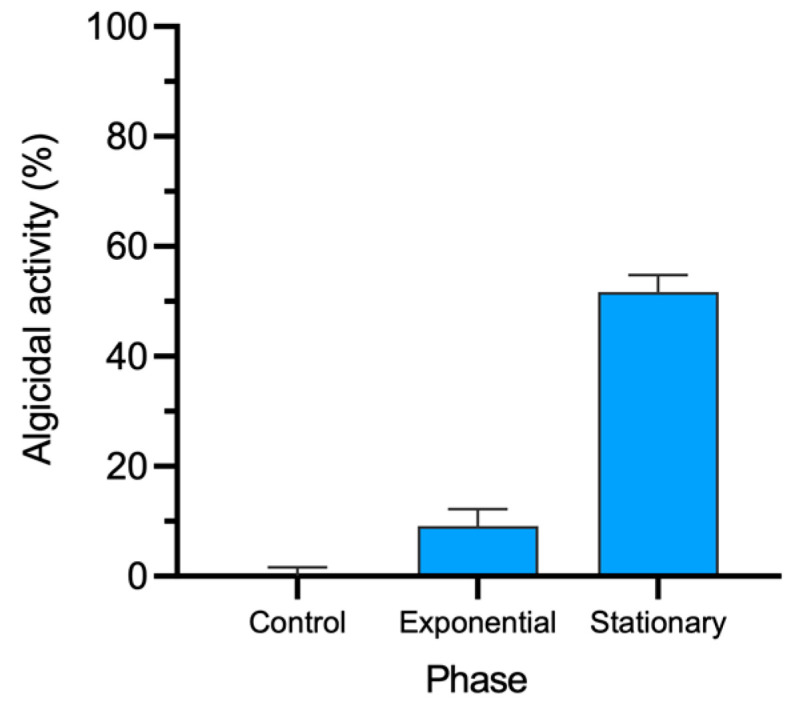
Antagonistic activity of the strain 0YLH supernatant against the dinoflagellate *P. triestinum* using CFS obtained from cultures in exponential and stationary growth phase.

The antagonistic activity against the dinoflagellates exposed to the strain 0YH supernatant was considerably higher when the CFS was obtained from bacterial cultures in the stationary phase (51.7% of algicide activity against *P. triestinum*) than when the CFS was obtained from the exponential phase (9.1% of algicidal activity) (Figure 3).

### 2.3. Stability of the Bacterial Supernatant of Strain 0YLH

**Figure 4:**
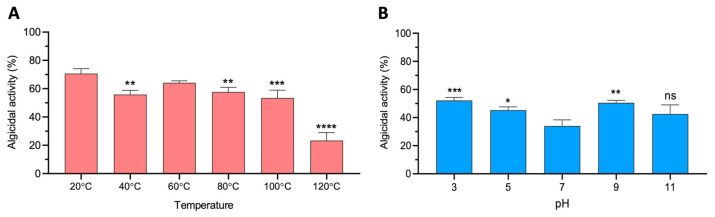
Stability of the supernatant of strain 0YLH when exposed to different (**A**) temperatures and (**B**) pH ranges. Asterisks denote statistical differences through one-way ANOVA and Dunnett test (ns *p* > 0.05; * *p* ≤ 0.05; ** *p* ≤ 0.01; *** *p* ≤ 0.001; **** *p* ≤ 0.0001).

To assess the stability of the CFS in different abiotic parameters, we evaluated the algicidal activity of the CFS in several conditions of temperature and pH. Our results showed that the CFS maintained algicidal activity over 40% against *P. triestinum* in a temperature range from 20 to 100 °C and a decrease to 20% when the CFS was incubated at 120 °C (Figure 4A). Additionally, the CFS showed remarkable algicidal activity under every pH condition tested, even when extreme pH values (3 or 11) were applied (Figure 4B).

### 2.4. Specificity of the Bacterial Supernatant of Strain 0YLH against Other Microorganisms

**Figure 5:**
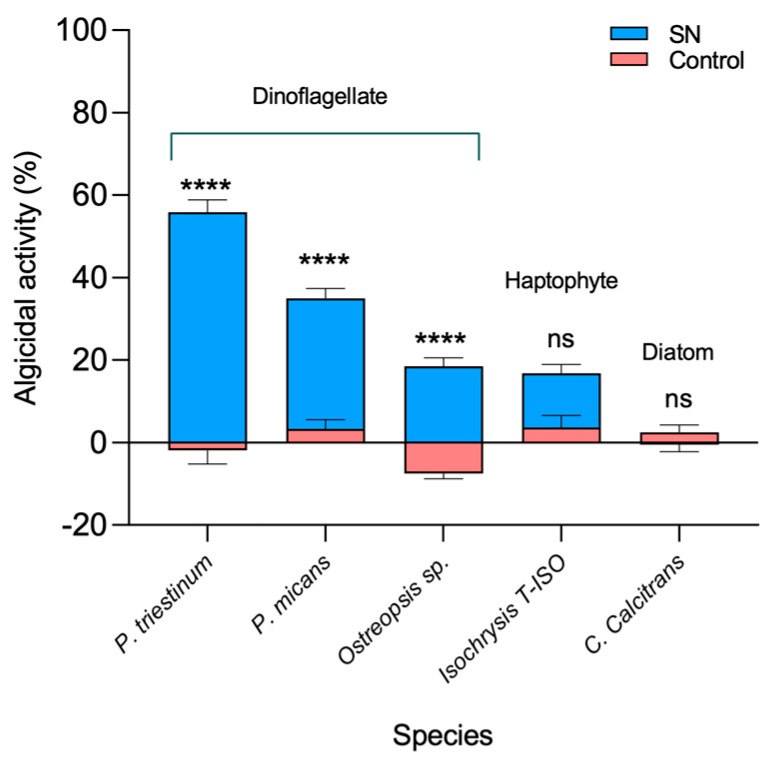
Algicidal activity of strain 0YLH against several dinoflagellate and diatoms after 24 h of exposure. Significant statistical differences (*p* ≤ 0.05) determined by two-way ANOVA are denoted by asterisks; ns represents no statistical difference.

Assessing the toxicity of the bacterial supernatant on other phytoplankton species is crucial for evaluating the potential use of this algicidal compound. Our results showed (Figure 5) that the algicidal effects were primarily observed in dinoflagellates. Notably, maximal effects were observed within the *Prorocentrum* genus, with 55.9% and 34.9% algicidal activity observed against *P. triestinum* and *P. micans*, respectively. The effect, although much weaker (18.5%), was also observed against the bentonic dinoflagellate *Ostreopsis* sp., and the haptophyte *Isochrysis* sp. T-ISO presented lower values (16.8% of algicide activity). Conversely, negative values were observed with the diatom *Cheatoceros calcitrans*.

## 3. Discussion

### 3.1. Characterization and Identification of 0YLH Strain

Our results showed that the bacterial isolate denoted as 0YLH belongs to *Shewanella halifaxensis*. Members of the *Shewanella* genera are found in many environments, such as water, soil and animal guts [24]. This clade possesses a close relationship to the *Alteromonas* genus, as observed in Figure 1. Several studies have reported that the *Alteromonas* and *Shewanella* clades might reach a high potential in marine biotechnology, since several isolates have already been used as algicidal compounds against HABs-forming organisms [25,26,27]. In this context, some remarkable considerations are that the *Shewanella* species have shown a versatile application against HABs and no adverse effects against non-harmful species, a characteristic which diminishes their negative environmental impacts [26,28]. Additionally, *Shewanella* species exudates have shown algicidal activity against diverse HAB-forming dinoflagellate species of the genera *Prorocentrum*, *Alexandrium* and *Karlodinium* [26,27,29]. Thus, the phylogenetic classification of the 0YLH isolate suggests potential applications of this bacterial isolate.

### 3.2. Algicidal Activity

Conversely to some previous studies which use the concentration of chlorophyll a (Chl a) to assess algicidal activity, our study used photosynthetic efficiency (Fv/Fm) [30,31]. In this context, the CFS demonstrated a high degree of algicidal activity against *P. triestinum*, a dinoflagellate previously described as a HABs-forming organism. We specifically measured the Fv/Fm values as an indicator of the photosynthetic state of dinoflagellates under bacterial CFS-induced stress. Normally, Fv/Fm values range between 0.5 and 0.7 [32,33,34]. When exposing the CSF of the 0YLH strain to the dinoflagellate *P. triestinum*, a decrease in Fv/Fm was observed. It is known that photosynthesis is essential for the physiological activities of microalgae, and also that bacteria with algicidal activity have been documented, which induce a dysfunction of the photosynthetic system. Therefore, microalgal lysis could be explained by structural changes in the chloroplasts, inhibiting the photosynthetic activity by decreasing the transport of electrons, the absorption of photons, and the malfunction of the PSII reaction center of the dinoflagellates, generating components that could damage cell integrity and finally induce death in dinoflagellates [21,35,36,37,38,39,40,41]. Additionally, PI staining was applied to determine cell viability, as this dye selectively enters damaged cells and binds to DNA molecules [42], resulting in fluorescence (Figure 2B); therefore, live cells remained unaffected by PI, indicating their intact membrane integrity, which was reflected by the absence of fluorescence [43].

There is previous evidence that bacterial media could interfere with antagonistic assays due to their high and diverse nutrient content [44]. To avoid these interferences, the bacterial culture medium used in the present work, i.e., f/2 medium supplemented with bactopeptone, was designed to avoid false positives and rule out any action of the bacterial culture medium on the dinoflagellate.

Additionally, in order to optimize CFS production, we evaluate the algicidal activity of the CFS obtained from 0YLH cultures obtained in the exponential and stationary phase of bacterial growth. Our results showed that algicidal activity was predominantly observed in the stationary phase. Thus, the increased algicidal potential in bacterial cultures during the stationary phase suggests that the bioactive effects may come from secondary metabolites excreted into the medium by strain 0YLH. These results are in agreement with earlier studies with other strains from the same genus, in which the algicide activity against dinoflagellates reached values of over 90% after 24 to 72 h exposure to CFS [27,45,46,47].

### 3.3. Stability of the Bacterial Supernatant of Strain 0YLH

The algicidal compound present in the supernatant was active even when exposed to high temperatures (i.e., thermostable). These results agree with previous studies showing a *Shewanella* sp. strain IRI-160 displaying algicide activity even after being subjected to sudden changes in temperature [46]. Regarding the pH stability of the CFS, our results showed that the CFS was stable in every tested condition even when extreme pH conditions, such as a pH of 3 or 11, were applied [47]. Considering the stability of the bioactive compound in such extreme conditions in the present study, we suggest that it is unlikely that the active compound is chemically similar to a peptide, protein or enzyme [48,49].

### 3.4. Specificity of the Bacterial Supernatant of Strain 0YLH against Other Microorganisms

The algicide activity of the CFS tested against several phytoplankton strains from our culture collection showed important differences between species. In Figure 5, maximal algicidal effects were observed on *P. triestinum*, a small-sized (~20 µm) planktonic dinoflagellate with a cellular wall dominated by two somehow delicate valve-like plates [50]. The effects were lower on *Ostreopsis* sp., a medium-sized benthic dinoflagellate with cell wall plates with a much coarser texture [51] and probably protected by the mucilaginous secretions used to get fixed in a substrate. Our hypothesis is that these differences in cell wall shape and texture may explain the distinct algicidal effect on the two genera (*Prorocentrum* and *Ostreopsis*). In addition, the effects on the haptophyte *Isochrysis* sp. T-ISO and the diatom *C. calcitrans* were low or even negative, which could indicate a slight stimulatory effect and could be due to nutrient regeneration by bacteria during incubation [52]. These results suggest a CFS specificity over bloom-producing organisms, since the supernatant mainly affected dinoflagellates of the genus *Prorocentrum*. Similar observations have been described in earlier studies with *Shewanella* and all the above supports the algicidal effect on the dinoflagellate species tested [52]. On the other hand, there are bacteria such as *Pseudoalteromonas* sp., which has activity on other dinoflagellates but not on the genus *Prorocentrum* [47]. Studies with other bacterial strains showed a similar effect against other microalgae groups, i.e., mild for *Isochrysis* and null for *Chaetoceros*, as in this study [53].

Algicidal bacteria have a significant impact on aquatic systems by influencing community dynamics through the release of metabolites into the surrounding environment, with the capability of lysing blooming algal species [20]. These novel compounds hold great potential as alternatives to traditional solutions or may act as complements, such as hydrogen peroxide, or physical methods including UV-C radiation, which are costly and lack specificity. As a result, biological agents have emerged as a more environmentally friendly solution, offering higher specificity in targeting harmful algal blooms (HABs) [54,55,56].

There are several reports of bacterial genera with algicide activity. For example, the genus *Acinetobacter* and its algicide compound 4-hydroxyphenethylamine directly affects the toxin secreted by the cyanobacterium *Microcystis aeruginosa* during the cyanobacterial harmful events (cyanoHAB events) of this species [57]. Another bacterium, *Alcaligenes denitrificans*, through a direct cell–cell interaction, causes cell lysis in the species of cyanobacteria *M. aeruginosa*, *M. viridis* and *M. wesenbergii*, and has a highly specific effect since it does not act against Chlorophyceae species [58]. Further, the *Bacillus thuringiensis* Q1 strain secretes a purine-derived compound, 2-[(2-Aceramido-6-oxo-6,9-dihydro-1H-purin-9-yl)methoxy], which has a negative effect on *M. aeruginosa* and *Anabaena flos-aquae* by inhibiting chloroplast formation [59]. Some other examples are listed, such as the bacterium *Deinococcus xianganensis* Y35 having an algicide effect on *Alexandrium tamarense* and the isolated compound identified as deinoxanthin [60], and *Pseudoalteromonas* sp. SP48 negatively affecting *A. tamarense*, damaging the organelle structures [61]. The *Pseudomonas* strain Ps3 effects algicidal against the species of red tide *Gymnodinium catenatum* and *Karenia mikimotoi* [62]. Another example is a *Vibrio* sp. isolate with antagonistic activity against the *Akashiwo* dinoflagellate, which through an over-production of reactive oxygen species (ROS), causes failures in the antioxidant system of the cells [63].

Bacteria of the genus *Shewanella* have been widely studied, are present throughout the world, both in freshwater and seawater, and are known for their versatility due to their various metabolic processes [64,65]. The *Shewanella* genus has a diversity of species that produce bioactive compounds with various effects, including antimicrobial activity against human and fish pathogens [66], antifungal activity by secreting volatile organic compounds [67], and algicidal activity by secreting highly specific compounds towards target microorganisms. For instance, *Shewanella* sp. Lzh-2 has algicidal effects on cyanobacterial blooms [37]. The *Shewanella* Y1 strain induces the lysis of *Alexandrium pacificum* due to the fact that it produces a deterioration in photosynthesis, inducing the overproduction of ROS and causing strong oxidative damage [27]. Another strain, *Shewanella* sp. IRI-160, activates rapid negative effects on harmful dinoflagellates, *Karlodinium veneficum*, *Karenia brevis*, *Gyrodinium instriatum*, *Cochlodinium polykrikoides*, *Heterocapsa triquetra*, *Prorocentrum minimal*, *Alexandrium tamarense* and *Oxyrrhis marina*, with a small hydrophilic molecule [29,38,46,52].

Overall, phytoplankton cells which are affected by algicide compounds of *Shewanella* genus have inhibitory photosystem II (PSII) effects, loss of transport of photosynthetic electrons, loss of cell membrane integrity and increased oxidative stress, i.e., causing damages at the DNA level and cell cycle interruption, eventually causing cell death [64,68].

Unfortunately, the isolation and characterization of bacterial algicidal compounds remain challenging, resulting in the limited availability of these bioactive fractions [69]. The purification and characterization processes possess difficulties, contributing to the scarcity of identified bacterial algicidal compounds. Notably, our results indicate high algicidal rates using the CFS, which is obtained from a process utilizing a low-nutrient culture medium, this being a feasible approach to obtain high volumes of CFS by scaling up the bacterial cultures.

## 4. Materials and Methods

### 4.1. Microalgal Strains and Culture Maintenance Conditions

*Prorocentrum triestinum*, strain ACIZ_LEM2 in the culture collection of the Centro de Bioinnovación de Antofagasta (CBIA), was isolated from a HAB event reported between November 2018 and February 2019 along the intertidal zone of San Jorge Bay in Antofagasta, Chile (23° S). Water samples were collected at two coastal points, “Puerto Costa” (−23.644; −70.399) and “Capilla Costa” (−23.686; −70.420). The isolation of *P. triestinum* was carried out by the single cell microcapillar pipetting method [70] under an inverted microscope (Olympus IX71, Turkey, Japan). The drops with the individually isolated cells were deposited in 1 mL microtubes with 5 μL of a modified f/2 medium [71] (HBO_3_ 1.86 M, NH_2_C(CH_2_OH)_3_ (Trizma solution) 250 mM, Na_2_SiO_3_*9H_2_O 105 µM). Cells were cultured photoautotrophically at a constant temperature of 21 °C and light intensity of 84–100 μmol m^−2^ s^−1^ (LED tubes). Cultures were maintained in exponential growth for subsequent experiments.

### 4.2. Bacterial Strain, Experimental Culture Conditions and Characterization

The bacterial strain 0YLH was isolated from the intertidal zone in San Jorge Bay, Antofagasta (23° S), at the time when *P. triestinum* was blooming, cultivated in 50 mL flasks of 120 mL with a modified f/2 medium supplemented with 0.2% of bactopeptone (*m*/*v*) and stirred in an orbital shaker (Thermolyne, Ramsey, MN, USA) at 120 rpm and 20 °C.

#### 4.2.1. DNA Isolation and Capillary Electrophoresis Sequencing (CES) of the 0YLH Strain

Genomic DNA (gDNA) was obtained with the PowerSoil^®^ MoBio extraction kit (MoBio Laboratories, Carlsbad, CA, USA). The concentration and purity of gDNA were evaluated using Nanodrop 2000c (Thermo Scientific, Waltham, MA, USA). The 16S rRNA gene was amplified using the primer pairs 27F, 518F, 800R and 1542R (27F, Forward: 5′-AGAGTTTGATCMTGGCTCAG-3′; 518F, Forward: CCAGCAGCCGCGGTAATACG, 800R, Reverse: TACCAGGGTATCTAATCC, 1492R, Reverse: TACGGYTACCTTGTTACGACTT). All samples were stored at −20 °C and sequenced by Macrogen Inc. (Seoul, South Korea) for CES services.

#### 4.2.2. Phylogenetic Analyses of Bacterial Strain 0YLH

The 16S rRNA sequences were trimmed and contigs were assembled using Geneious Prime software (v 2022.2.2; [72]). The phylogenetic tree was developed with jModelTest-2 software (v2.1.10; [73]) and the Tamura-Nei nucleotide substitution model [74], applying 100 bootstraps for the phylogenetic tree. The target sequence was labeled as “0YLH” and aligned against 50 members of the Alteromonadales order. The outgroup member used was *Photobacterium damselae* (Accession number MT071398). The Maximum Likelihood algorithm PhyML [75] was used on the alignment previously obtained with the MUSCLE algorithm [76]. All assembled sequences obtained were deposited in the GenBank under the accession number OR025894.

### 4.3. Production of 0YLH CFS for Evaluation of Algicidal Effects

The CFS of strain 0YLH was obtained by culturing the bacterium until reaching the stationary growth phase, at a McFarland standard density of 5. The culture was then centrifuged at 10,000 rpm for 10 min, and the supernatant was filtered through sterile Minisart PES filters 28 mm for syringe and 0.22 µm pore size (Sartorius, Gottingen, Germany).

### 4.4. Algicidal Activity

The algicide assays were performed in 6-well culture plates, and each well was filled with 8 mL of *P. triestinum* culture in the exponential phase with an initial cell density of 10^4^ cells mL^−1^, and 2 mL of the CFS of the 0YLH strain (i.e., a final concentration of 20% *v*/*v*). In addition, an equivalent volume of water enriched with modified f/2 medium + BP was used as a negative control. All assays were performed in triplicate. The experiment was carried out for 24 h; cell densities in *P. triestinum* cultures were estimated from cell counts with an inverted microscope (Olympus IX71, Japan). The maximum potential quantum efficiency of Photo system II (PSII) (Fv/Fm) was measured through the AquaPen-C fluorometer (Photon Systems Instruments, Drásov, Czech Republic). To test cell viability, 1 mL of culture aliquots was centrifuged at 4000 rpm for 1 min, 800 µL of the supernatant was removed and 2 µL of 0.014 mM PI stain was added to the remaining 200 µL and incubated for 1 h at room temperature in the dark. Images were taken with a ZOE Fluorescent Cell Imager (BIO-RAD, USA). The algicidal activity of the 0YLH strain was calculated using the following equation:

$$Algicidal\;activity\;(\%)=\frac{(N_{0}-N_{t})\;}{N_{0}}\times 100$$

where *N_t_* and *N*_0_ are the values of Fv/Fm of the microalgae at time *t* and time 0 (start of assay), respectively.

The CFS specificity was tested against microalgae species belonging to different groups, including two dinoflagellates (*Prorocentrum micans* and *Ostreopsis* sp.), one haptophyte (*Isochrysis* sp. T-ISO) and one diatom (*Chaetoceros calcitrans*).

### 4.5. Stability of the Bacterial Supernatant

CFS aliquots were incubated at 20 °C, 40 °C, 60 °C, 80 °C, 100 °C and 120 °C for 2 h to test the effects of temperature on the algicidal activity. Furthermore, the pH of the CFS aliquots was adjusted to 3, 5, 7, 9 and 11 to assess the pH tolerance of the algicidal compound, with a pH of 7 being the control. Each treated supernatant was subsequently inoculated into *P. triestinum* cultures in triplicate at a final concentration of 20% (*v*/*v*) to test the algicide activity. Aliquots of f/2 medium supplemented with bactopeptone were used as a negative control.

### 4.6. Statistical Analysis

All tests were conducted in triplicate, and all statistical analyses were performed using GraphPad PRISM software (Version 9.2.0; GraphPad Software, Inc., San Diego, CA, USA). One-way or two-way ANOVA and Dunnett test analyses were used. Confidence levels of 95% and *p* values ≤ 0.05 were considered statistically significant.

## 5. Conclusions

The classified *Shewanella halifaxensis* 0YLH strain isolated from Antofagasta Bay, Chile, demonstrated a robust indirect algicidal effect on the dinoflagellate *P. triestinum*. The exposure of *P. triestinum* to the filtrate of the CFS, obtained from centrifuged and filtered bacterial culture at the stationary phase, disrupted the dinoflagellate photosynthetic efficiency, leading to cell stress and eventual cell lysis. Notably, the algicidal compound exhibited a remarkable stability against high temperatures and pH variations.

Our research confirms that the 0YLH strain shows algicidal activity against *P. triestinum* blooms, with some level of specificity towards this genus. Consequently, it represents a promising candidate for mitigating harmful algal blooms caused by these dinoflagellates. Furthermore, it is essential that future studies isolate and identify the specific bioactive compounds responsible for the algicidal activity from the supernatant or other exudates, as they hold significant potential for future applications.

## Data Availability

Not applicable.
